# Community oncologists’ perceptions and utilization of large-panel genomic tumor testing

**DOI:** 10.1186/s12885-021-08985-0

**Published:** 2021-11-25

**Authors:** Eric C. Anderson, Alexandra C. Hinton, Christine W. Lary, Anny T. H. R. Fenton, Andrey Antov, Emily Edelman, Petra Helbig, Kate Reed, Susan Miesfeldt, Christian A. Thomas, Michael J. Hall, J. Scott Roberts, Jens Rueter, Paul K. J. Han, MCGI Working Group, Nicholette Erickson, Mayur Movalia, Marek Skacel, Allan Espinosa, Ridhi Gupta, Rachit Kumar, Richard Polkinghorn, Christopher Darus, Scot Remick, Robert Christman, Karen Rasmussen, Christian A. Thomas, Philip Brooks, Catherine Chodkiewicz, Antoine Harb, Sarah Sinclair, Peter Rubin, Elizabeth Connelly, Peter Georges, Jennifer Bourne, Linda Choquette, Ken Fasman, Cristen Flewellen, Emily Edelman, Lory Guerrette, Petra Helbig, Susan Mockus, Kate Reed, Jens Rueter, Kunal Sanghavi

**Affiliations:** 1 Center for Outcomes Research and Evaluation, Maine Medical Center Research Institute, Portland, ME USA; 2 Tufts University School of Medicine, Boston, MA USA; 3 The Jackson Laboratory, Augusta, ME USA; 4 Maine Medical Center, Portland, ME USA; 5 New England Cancer Specialists, Scarborough, ME USA; 6 Fox Chase, Philadelphia, PA USA; 7 University of Michigan School of Public Health, Ann Arbor, MI USA

**Keywords:** Genomic, Cancer, Uncertainty, Attitudes, Confidence

## Abstract

**Purpose:**

Large-panel genomic tumor testing (GTT) is an emerging technology with great promise but uncertain clinical value. Previous research has documented variability in academic oncologists’ perceptions and use of GTT, but little is known about community oncologists’ perceptions of GTT and how perceptions relate to clinicians' intentions to use GTT.

**Methods:**

Community oncology physicians (*N* = 58) participating in a statewide initiative aimed at improving access to large-panel GTT completed surveys assessing their confidence in using GTT, attitudes regarding the value of GTT, perceptions of barriers to GTT implementation, and future intentions to use GTTs. Descriptive and multivariable regression analyses were conducted to characterize these perceptions and to explore the relationships between them.

**Results:**

There was substantial variability in clinicians’ perceptions of GTT. Clinicians generally had moderate confidence in their ability to use GTT, but lower confidence in patients’ ability to understand test results and access targeted treatment. Clinicians had positive attitudes regarding the value of GTT. Clinicians’ future intentions to use GTT were associated with greater confidence in using GTT and greater perceived barriers to implementing GTT, but not with attitudes about the value of GTT.

**Conclusions:**

Community oncologists’ perceptions of large-panel genomic tumor testing are variable, and their future intentions to use GTT are associated with both their confidence in and perceived barriers to its use, but not with their attitudes towards GTT. More research is needed to understand other factors that determine how oncologists perceive and use GTT in clinical practice.

**Supplementary Information:**

The online version contains supplementary material available at 10.1186/s12885-021-08985-0.

## Introduction

Genomic tumor testing is an emerging technology that promises to improve cancer treatment outcomes and has already enabled successful targeted, “precision” treatments for common and difficult to treat cancers. Prominent examples include erlotinib for EGFR-mutant lung cancer [1] and vemurafenib for BRAF V600E–mutant melanoma [2]. Tumor tests for mutations with known FDA-approved treatments have quickly become the standard of care. In recent years, next-generation genome sequencing technology has enabled the development of large-panel genomic tumor tests (GTT) that can test and identify variants in hundreds of genes simultaneously. However, for many variants identified by large-panel GTT no current FDA-approved treatments exist, and the clinical utility of these extended, large-panel testing is debatable. Nevertheless, GTT is increasingly utilized in clinical practice. In a 2017 study, 75.6% of oncologists reported currently using multi-gene GTT to guide treatment decisions [3]. Moreover, as genomic technologies become less expensive and more accurate [4], utilization of GTT will likely increase and continue to identify more genomic variants with uncertain clinical utility [5–7].

These associated uncertainties, along with the increasing dissemination and implementation of GTT in clinical practice, make it important to understand how oncologists perceive, understand, and actually use this technology. Past research on this topic, however, has been limited and focused primarily on academic oncologists’ confidence in using GTT. Gray et al. conducted a single-institution study of academic medical oncologists, and found variability in clinicians’ confidence in their knowledge of GTT and their ability to both explain GTT to patients and to make treatment recommendations based on genomic information [8]. More recent studies have yielded mixed findings regarding physicians’ understanding and confidence in their ability to interpret, use, and discuss the results of GTT with patients [9, 10]. These mixed findings may reflect differences in the clinical settings, disease types, and sample populations of these studies, and raise the need for further research.

More research is also needed to investigate how clinicians’ perceptions of GTT influence their utilization of these tests. Gray et al. demonstrated that greater confidence in the use of GTT among oncologists was associated with greater anticipated future use of GTT [8]. This finding is consistent with theories of health behavior, which theorize that confidence—also referred to as self-efficacy [11]—is a critical determinant of health behavior. However, past studies of GTT have not investigated the potential influence of other factors thought to be equally critical, including attitudes regarding the value of GTT and perceptions of barriers to GTT utilization. Most research on clinicians’ perceptions of GTT has also been limited to oncologists practicing at large academic medical centers (e.g. [8, 9, 12],); clinicians practicing in community-based settings and rural areas have received less attention. Emerging evidence suggests, however, that community oncologists may use GTT less frequently [13] and that oncologists in rural areas might have more limited genomic knowledge [14]. More research is thus needed to characterize the knowledge, attitudes, and practices of community oncologists in more rural settings, given that most cancer care in the US is provided in such settings, and many rural communities have disproportionately high cancer incidence and mortality rates [15].

The objective of this study was to understand community oncology clinicians’ perceptions of GTT and how they relate to clinicians’ intentions to use GTT. We focused on three types of perceptions theoretically related to GTT use: confidence (self-efficacy) regarding the use of GTT; attitudes regarding the value of GTT; and perceived barriers to implementing GTT (e.g. cost, incidental germline findings). Major health behavior theories hold that behavioral intentions are key precursors of actual health behaviors [16, 17]. We therefore treated future intentions to use GTT as a proxy for actual test-ordering behavior, in order to explore how clinicians’ various perceptions of GTT might be related to their use of the test.

This study leveraged a unique program, the Maine Cancer Genomics Initiative (MCGI), designed to overcome the implementation hurdles for large-panel GTT and precision oncology in rural community settings. The MCGI is a ten-year (2016–2026), longitudinal, statewide, multi-site initiative aimed at disseminating GTT in community oncology practices throughout the State of Maine. The MCGI provides clinicians and their patients with access to free large-panel GTT as well as clinician education and decision support services, including genomic tumor boards conducted at multiple practice sites throughout the state. The MCGI thus provided a unique opportunity to assess how community-based oncologists in a predominantly rural state perceive and use GTT in their practices.

## Methods

### Study population and design

The study population consisted of actively practicing oncology physicians in Maine, including hematologists/oncologists, gynecologic oncologists, and surgical oncologists. Physicians were recruited by the MCGI research team via in-person site visits, email, and telephone. Informed consent was obtained from all participants. Upon joining the MCGI, participating clinicians completed a 30-min baseline survey containing a variety of measures, including sociodemographic and practice information, as well as clinicians’ perceptions and future intentions to use GTT. The survey was self-administered using the online survey platform RedCap Cloud™ between June 2017 and October 2018. The MCGI study protocol was reviewed and approved by the Western Institutional Review Board. This study was performed in accordance with the ethical standards of the Declaration of Helsinki or comparable ethical standards.

### Measures

The survey questionnaires contained items measuring the following constructs (see supplemental online Table 1 for exact wording and response options for each survey question). Questionnaire items were developed by adopting or adapting existing measures in the literature, as noted below, or developing new items which were piloted among 3 practicing medical oncologists and further refined by our team.

### Future intentions to use GTT

Clinicians’ intentions to use GTT in the future were measured by a single question, similar to an item used by Gray et al. [8], which asked clinicians how many GTTs they anticipated ordering in the next 12 months.

### GTT-related confidence

**Fig. 1:**
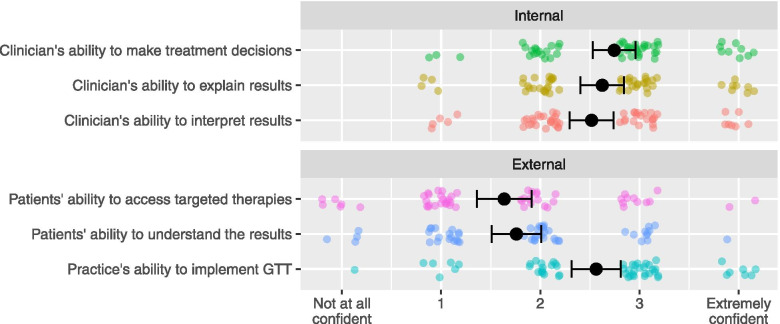
GTT-related Confidence. Black dots represent means. Error bars represent standard error of the mean. Colored dots represent each participant’s response (position jittered to avoid overplotting). The x-axis labels reflect the response labels on surveys questions: 0 = not at all confident to 4 = extremely confident (intermediate options were not labelled)

Two aspects of clinicians’ confidence in using GTT were assessed (Fig. 1). First, clinicians’ confidence or self-efficacy regarding their own ability to use GTT—which we designate “internal confidence”—was assessed by three questions, adapted from Gray et al. [8] The questions asked clinicians to rate their confidence in their ability to perform three tasks: (1) interpreting test results; (2) explaining test results to patients; and (3) using results to inform treatment decisions (Cronbach’s α = 0.877). Second, clinicians’ confidence in the ability of other stakeholders (including their practice and patients) to use GTT—which we designate “external confidence”—was assessed by three questions asking clinicians to rate their confidence in: (1) their practice’s ability to implement GTT; (2) their patients’ ability to understand GTT results; and (3) their patients’ ability to access targeted therapies and clinical trials (Cronbach’s α = 0.698). All answers utilized a 5-point Likert scale; 0 = not at all confident to 4 = extremely confident. For each construct, a summary score was calculated by averaging answers.

### Attitudes towards GTT

**Fig. 2:**
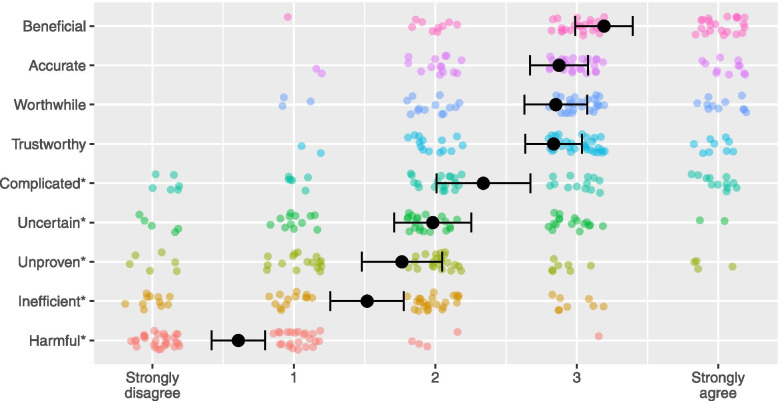
Attitudes about the value of GTT. Legend: * = items were reverse coded for aggregated measure and modeling. Black dots represent means. Error bars represent standard error of the mean. Colored dots represent each participant’s response (position jittered to avoid overplotting). The x-axis labels reflect the response labels on surveys questions: 0 = strongly disagree to 4 = strongly agree (intermediate options were not labelled)

Clinicians’ attitudes regarding the value of GTT were assessed by 9 newly developed questions that our team adapted from attitudinal measures and findings of prior studies of physician and patient attitudes towards genomic testing [8, 18, 19]; the same questions were also asked of patient participants in this study. The questions asked clinicians to rate their agreement with 4 positively and 5 negatively valenced attitudes (Fig. 2). The questions began with the statement, “Genomic tumor testing seems …” which was then paired with different adjectives (beneficial, harmful*, uncertain, accurate, trustworthy, unproven*, complicated*, inefficient*, and worthwhile). Ratings utilized a 5-point Likert response scale; 0 = strongly disagree to 4 = strongly agree. Negative items (identified above with an *) were reverse coded so that higher values corresponded to more positive attitudes. A summary score was calculated by averaging answers (Cronbach’s *α* = 0.698).

### Perceived barriers to GTT implementation

**Fig. 3:**
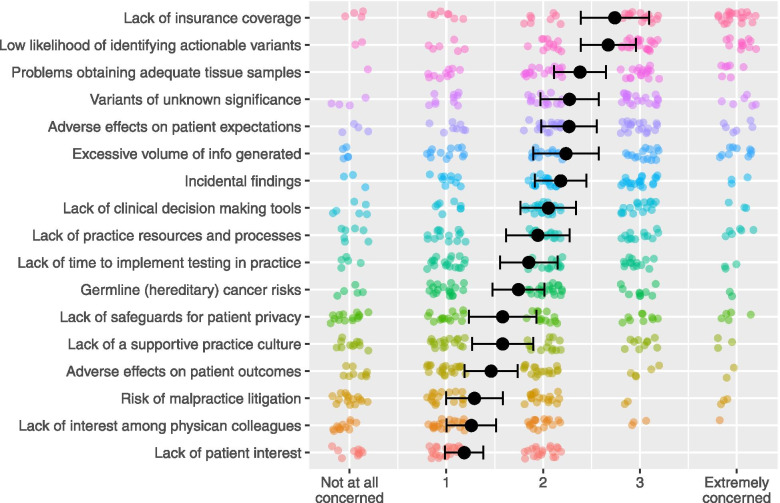
Perceived Barriers to GTT Implementation. Legend: Black dots represent means. Error bars represent standard error of the mean. Colored dots represent each participant’s response (position jittered to avoid overplotting). The x-axis labels reflect the response labels on surveys questions: 0 = not at all concerned to 4 = extremely concerned (intermediate options were not labelled)

Clinicians’ perceptions of barriers to implementing GTT in clinical practice were assessed by 17 questions that our team developed based on barriers identified in prior physician studies (Fig. 3) [9]. For each of these items, clinicians were asked to rate their concerns about the use of GTT in their own practice using a scale of 0 = not at all concerned to 4 = extremely concerned. A summary score was calculated by averaging answers (Cronbach’s *α* = 0.832).

### Data analysis

Descriptive statistics were generated to characterize the study population. Internal consistency reliability for the clinician perceptions and attitudes measures was assessed by calculating Cronbach’s *α* coefficient. Next, we fit linear regression models to identify factors associated with clinicians’ intentions to order GTT in the future. We first modeled clinician sociodemographic and practice characteristics (gender, years since medical school, practice size, rurality, and patient volume) alone, then added behavioral variables (summary scores for confidence, attitudes, and perceived barriers) to the model. The distribution of the outcome variable (number of GTTs clinicians intend to order) was right-skewed; therefore, this variable was log-transformed for all models to meet normality assumptions. A value of 1 was added to all values to allow for zero values to be log-transformed. Coefficients reported here were transformed back (exponentiated) to correct for the log-transformation, allowing the coefficients to be more easily interpreted. All analyses were conducted using R 3.5.3 [20].

## Results

The study team identified 68 oncology physicians in Maine who were invited to join the Maine Cancer Genomics Initiative as of October, 2018. One physician was not currently engaged in clinical practice and was excluded from this analysis. Of the remaining physicians, 58 (87%) joined, consented to participate, and completed the survey (Table 1). The sample contained approximately equal number of women (48%) and men (52%), with an average of 19 years of practice since medical school (range = 4–45 years). The majority of participants were hematology/oncology specialists (84%), and a substantial proportion (62%) practiced in rural or small town settings. The average number of newly diagnosed patients seen for treatment evaluation each month was 21 (range = 5–45).

**Table 1:** Physician Demographic and Practice Variables

N (%)	
**Gender**	
*Female*	26 (48%)
*Male*	28 (52%)
*Missing*	4
**Rurality**	
*Rural*	18 (34%)
*Small town*	15 (28%)
*Suburban*	12 (23%)
*Urban*	8 (15%)
*Missing*	5
**Specialty**	
*Hematology/Oncology*	48 (84%)
*Surgical Oncology*	4 (7.0%)
*Gynecologic Oncology*	2 (3.5%)
*No Specialty Identified*	3 (5.3%)
*Missing*	1
**Practice Size (Number of oncology physicians)**	
*1–4*	23 (45%)
*5–9*	12 (24%)
*10+*	16 (31%)
*Missing*	7
**Years since medical school**	
*1–9*	8 (15%)
*10–19*	24 (44%)
*20–29*	13 (24%)
*30+*	9 (17%)
*Missing*	4
**Mean (SD)**	
**Average number of newly diagnosed patients each month**^a^	21 (10)
**Percentage of time spent on direct patient care**^b^	88% (13)
**Patient Insurance Status (percent of caseload):**	
Uninsured^c^	12% (9)
Medicaid^d^	22% (13)
Commercially Insured^e^	27% (10)
Medicare^f^	44% (13)

Number of responses: ^a^n = 52; ^b^n = 52;^c^n = 47; ^d^n = 47; ^e^n = 48; ^f^n = 47

### Clinicians’ perceptions of GTT

Clinicians had a generally high but variable confidence in their own ability to effectively utilize GTT (Fig. 1), and relatively low confidence in their patients’ ability to understand GTT results and access targeted therapies (Fig. 1, Supplemental Table S1).

Despite their varying levels of internal and external confidence, clinicians generally had positive attitudes regarding the value of GTT. They showed high levels of agreement that GTT is beneficial, worthwhile, accurate, and trustworthy, and high levels of disagreement that GTT is harmful, causes issues with patient privacy, or is inefficient. There was variation in their level of agreement that GTT is uncertain, complicated, and can help most patients (Fig. 2, Supplemental Table S1).

Perceived barriers to GTT use also varied widely across clinicians. In general, clinicians were most concerned about the logistics of implementing GTT and the low likelihood of identifying clinically actionable results, and least concerned about the lack of patient interest, litigation, and patient privacy (Fig. 3, Supplemental Table S1).

### Factors associated with intentions to use GTT

Clinicians reported intentions to order an average of 26 GTTs in the next 12 months (range 0–150). As noted above, the distribution was right-skewed with a few clinicians ordering a large number of tests.

Multivariable regression analysis including only clinician sociodemographic characteristics in the model showed no significant associations with GTT intentions (Supplemental Table S2). However, in the regression model including both clinician sociodemographic and psychological variables, future intentions to use GTT were significantly higher for clinicians with more practice experience (greater years since medical school): b = 1.04; 95% CI 1.01, 1.07; *p* = 0.020 (Table 2). Future intentions to use GTT were also significantly associated with greater internal confidence (i.e., their own ability to utilize GTT) (b = 2.07; 95% CI 1.24, 3.48; *p* = 0.007; Table 2), and with greater perceived barriers (b = 1.88; 95% CI 1.07, 3.32; *p* = 0.030). Future intentions were not significantly associated with attitudes regarding the value of GTT or external confidence (i.e., patients ability to understand results or access treatment and their practice’s ability to implement GTT) though there was a non-significant trend for both associations (Table 2).

**Table 2:** Predictors of Intentions to use Genomic Tumor Tests in the Next 12 Months

	Factor^**1**^	95% CI^***2***^	***p***-value
**Demographic Variables**			
Gender			
Female	–	–	
Male	1.72	0.98, 3.03	0.061
Years since medical school	1.04	1.01, 1.07	0.020
Practice Size (Number of oncology physicians)	0.99	0.92, 1.07	0.8
Rural practice location	0.66	0.34, 1.26	0.2
Average number of newly diagnosed patients each month	1.01	0.98, 1.05	0.3
**Psychological Variables**			
Attitudes Summary Score	1.79	0.87, 3.68	0.11
Confidence Summary Score - Internal	2.07	1.24, 3.48	0.007
Confidence Summary Score - External	0.64	0.38, 1.08	0.094
Barriers Summary Score	1.88	1.07, 3.32	0.030

^1^ Factor = exponetiated regression coefficient; every unit increase in the predictor variable is associated with a multiplicative effect of the coefficient on the number of GTT orders in the next 12 months

^2^ *CI* Confidence Interval

N = 48 due to some participants were missing data (see Table 1)

## Discussion

This study examined community-based oncology physicians’ perceptions of GTT and how those perceptions relate to their intentions to use GTT in clinical practice. To our knowledge, this is the first study to include oncologists practicing in rural community settings, and to assess not only oncologists’ confidence in using GTT but also their attitudes towards GTT use, and their perceived barriers, and to examine how these factors relate to their future use of GTT. The study yielded several findings that have important implications for the implementation of GTT in community oncology practice settings.

First, we found that more years since medical school was associated with greater intentions to order GTTs. This finding contrasts a nationally representative study which suggested that oncologists were more likely to order GTTs if they were younger than 50 years old [3]. This discrepancy may be due to differences in measures (e.g., dichotomized age vs years since medical school) and study populations. Interestingly, although a small study in Canada demonstrated rural-urban differences in genomic expertise and knowledge [14], our study showed no effect of rurality; however, our entire sample was arguably more rural than populations examined in other studies.

Consistent with previous research in academic physician populations [8, 12], we found wide variation in community-based oncologists’ confidence in using GTT. Clinicians in our study reported generally high levels of confidence in their own ability to use GTT, and lower confidence in patients’ ability to understand results and access targeted therapy. Although our goal was not to directly compare levels of confidence to those observed in other studies, when we rescaled rating of confidence to a shared 0–1 scale (0 = low confidence to 1 = high confidence), clinicians in our study had very similar confidence in their own abilities (Mean = .655) compared to clinicians in Gray et al. (Mean = .667; see supplemental online materials for additional details) [8]. Also consistent with findings from Gray et al. [8] and as predicted by theories of health behavior [16, 17], we found that greater clinician confidence in their ability to use GTT (i.e., self-efficacy) was associated with greater future intentions to use GTT. Together, these findings suggest that the successful dissemination and implementation of GTT in community oncology settings might depend on increasing oncology clinicians’ confidence in using GTT, in addition to ensuring that patients are able to benefit from GTT. Clinicians’ internal confidence in GTT might be increased through various educational interventions, including genomic tumor boards, while their external confidence might be increased through the provision of patient-focused decision support or navigation services.

We also found that community-based oncologists had generally positive attitudes regarding the value of GTT. In health behavior theories, attitudes are important determinants of behavior [21–23]. Interestingly, however, in our study attitudes were not associated with clinicians’ intentions to order GTT. More research is needed to explain this lack of association, but one possibility is that other variables—such as local practice norms or the availability of GTT—may have greater influence on clinicians’ intentions and actual use of GTT. The relative lack of variation in clinician attitudes towards GTT in our sample may also have contributed to the observed lack of association between GTT attitudes and intentions.

Our study also found wide variation in community-based oncologists’ perceptions of barriers to utilizing GTT. Overall, the barriers of greatest concern were lack of insurance coverage and low probability of finding actionable results. It is interesting that lack of insurance coverage was considered a barrier of concern since the MCGI initiative offered free testing. Most likely, this concern existed before MCGI, and may return after the initiative ends. The barriers of least concern were lack of patient and colleague interest. Variants of unknown significance and managing patient expectations were barriers of moderate concern, mirroring findings using qualitative interviews [9]. Clinicians in our study also perceived incidental identification of germline mutations as a barrier of moderate concern, similar to a study of patients [24]. Paradoxically, we found greater overall level of clinician concern about barriers to GTT was associated with greater intentions to use GTT. A possible explanation of this finding is that the level of concern about barriers to GTT may simply be a marker or result of greater use of GTT, rather than a cause. This finding raises the need for further research studies, using longitudinal or experimental designs, that can establish the causal direction of these and other relationships. In any case, future efforts to disseminate and implement GTT in community oncology practice will need to address the perceived and real barriers to its use.

### Limitations, strengths, and future directions

This study had several limitations that qualify the findings and call for further research. First, the study was conducted as part of a broader implementation initiative that offered free GTT along with educational support (e.g. genomic tumor boards). Both of these factors may have influenced clinicians’ perceptions and use of GTTs, and may limit the generalizability of our findings to other practice settings. Furthermore, our study sample was relatively small and limited to a single state. For instance, we lacked power to explicitly test for differences between rural and urban practices, an important question that will need to be addressed by future studies. Additionally, future work should test whether other variables, like the proportion of time clinicians spend on patient care, influence test ordering. Nevertheless, to our knowledge the current study is the first of its kind to enroll nearly all practicing community oncology physicians in a predominantly rural state, and was thus regionally representative. The current study was cross-sectional in nature; therefore, we cannot draw inferences about the causal directions of the observed associations. However, our study generates hypotheses that can be tested in more definitive future studies, which we will be conducting in the future using longitudinal data that is currently being collected. The current study also used intentions to order GTT as a proxy for actual GTT use; our future studies will address this limitation as well, by examining actual test-ordering behavior. Finally, because GTT is a relatively new intervention in cancer care, many of our study measures were newly developed, and further research is needed to assess their reliability and validity. Despite these limitations, our study provides important new evidence on community-based oncology physicians’ perceptions and practices regarding GTT, and the relationships between them.

## Conclusions

Community oncologists’ perceptions of GTT vary widely, and their confidence in their ability to use GTT is associated with their future intentions to order it. A better understanding of these factors will enable clinicians, researchers, and health policy makers to address barriers to disseminating and implementing GTT in community oncology practice and to promote its appropriate use.

## Supplementary Information

**Additional file 1.**

## Data Availability

The dataset from the current study is not publicly available because the small sample of clinicians might be re-identifiable. A limited version of the dataset is available from the corresponding author on request.

## Abbreviations

GTT: Large-panel Genomic tumor test
MCGI: Maine Cancer Genomics Initiative
