# Supplementary material for: Community oncologists’ perceptions and utilization of large-panel genomic tumor testing
Source: BMC Cancer. 2021 Nov 25;21:1273. doi: 10.1186/s12885-021-08985-0 (PMC8620967; doi:10.1186/s12885-021-08985-0)
Supplement: Supplementary file 1 — Additional file 1. [file 12885_2021_8985_MOESM1_ESM.docx]

**Supplemental Online Materials**

In the discussion, we report comparing confidence ratings in our study to findings by Gray et al. ^1^ The two studies used different scales, making direct comparison of raw values impossible (our study used 0-4 scale while Gray used a 1-4 scale). To make a comparison across the two studies, we rescaled rating of confidence from each study to a shared 0-1 scale (0 = low confidence to 1 = high confidence). This rescaling was done by taking the score, subtracting the lowest value on the scale, and then dividing by the range.^2^ So for the mean confidence in the Anderson study that was: (2.62-0)/4 = 0.655. The mean confidence in the Gray study was (3-1)/3 = 0.677.

**Table S1. Summary statistics: Genomic Tumor Test (GTT) Use, and Confidence, Attitudes, and Barriers.**

| **Characteristic*^1^*** | **N** | **Mean(SD)** |
| --- | --- | --- |
| **Use of genomic tumor testing** | | |
| Number of GTT you plan to order in next 12 months | 56 | 26 (33) |
| **Please rate how confident you feel about the following:** | | |
| ***Internal Confidence*** | | |
| Your ability to interpret the results of GTT | 56 | 2.52 (0.83) |
| Your ability to explain the results of GTT to patients | 56 | 2.62 (0.82) |
| Your ability to make appropriate treatment decisions based on GTT | 55 | 2.75 (0.80) |
| Summary Score: Internal Confidence | 56 | 2.62 (0.75) |
| ***External Confidence*** | | |
| Your practice’s ability to implement GTT | 55 | 2.56 (0.92) |
| Your patients’ ability to understand the results of GTT | 54 | 1.76 (0.91) |
| Your patients’ ability to access targeted therapies and/or clinical trials identified by GTT | 55 | 1.64 (1.02) |
| Summary Score: External Confidence | 56 | 2.18 (0.65) |
| **Attitudes: Genomic tumor testing seems:** | | |
| Beneficial | 57 | 3.19 (0.77) |
| Harmful | 56 | 0.61 (0.71) |
| Uncertain | 56 | 1.98 (1.02) |
| Accurate | 56 | 2.88 (0.76) |
| Trustworthy | 55 | 2.84 (0.74) |
| Unproven | 55 | 1.76 (1.05) |
| Complicated | 56 | 2.34 (1.24) |
| Inefficient | 56 | 1.52 (0.97) |
| Worthwhile | 54 | 2.85 (0.81) |
| Summary Score: Attitudes | 56 | 2.48 (0.46) |
| **Barriers: How concerned are you about the following potential problems with genomic tumor testing?** | | |
| Excessive volume of info generated | 55 | 2.24 (1.25) |
| Low likelihood of identifying actionable variants | 55 | 2.67 (1.06) |
| Problems obtaining adequate tissue samples | 55 | 2.38 (0.99) |
| Incidental findings | 55 | 2.18 (0.98) |
| Variants of unknown significance | 55 | 2.27 (1.11) |
| Germline (hereditary) cancer risks | 55 | 1.75 (0.99) |
| Adverse effects on patient outcomes | 54 | 1.46 (1.00) |
| Adverse effects on patient expectations | 56 | 2.27 (1.07) |
| Risk of malpractice litigation | 55 | 1.29 (1.08) |
| Lack of patient interest | 54 | 1.19 (0.73) |
| Lack of interest among physician colleagues | 54 | 1.26 (0.94) |
| Lack of time to implement testing in practice | 54 | 1.85 (1.09) |
| Lack of practice resources and processes | 55 | 1.95 (1.21) |
| Lack of a supportive practice culture | 55 | 1.58 (1.17) |
| Lack of clinical decision making tools | 55 | 2.05 (1.06) |
| Lack of insurance coverage | 54 | 2.74 (1.29) |
| Lack of safeguards for patient privacy or confidentiality | 55 | 1.58 (1.29) |
| Summary Score: Barriers | 56 | 1.93 (0.56) |
| *^1^*Statistics presented: mean (SD) | | |

**Table S2. Demographic Predictors of the Number of Genomic Tumor Test Orders in the Next 12 Months**

|  | **Factor^1^** | **95% CI*^2^*** | **p-value** |
| --- | --- | --- | --- |
| Gender |  |  |  |
| Female | — | — |  |
| Male | 1.74 | 0.95, 3.19 | 0.072 |
| Years since medical school | 1.02 | 0.99, 1.05 | 0.2 |
| Practice Size (Number of oncology physicians) | 0.98 | 0.91, 1.06 | 0.6 |
| Rural practice location | 1.00 | 0.52, 1.94 | >0.9 |
| Average number of newly diagnosed patients each month | 1.02 | 0.99, 1.06 | 0.2 |
| *^1^Factor = exponetiated regression coefficient; for every unit increase in the predictor variable, there is a multiplicative effect of the coefficient on the number of GTT orders in the next 12 months.*  ^2^ CI = Confidence Interval  N = 49 due to some participants were missing data (see Table 1). | | | |

**References**

1. Gray SW, Hicks-Courant K, Cronin A, Rollins BJ, Weeks JC. Physicians’ attitudes about multiplex tumor genomic testing. *J Clin Oncol*. 2014;32(13):1317-1323. doi:10.1200/JCO.2013.52.4298
2. Giannoulis C. Rescaling Sets of Variables to Be on the Same Scale. The Analysis Factor Accessed October 22, 2021. https://www.theanalysisfactor.com/rescaling-variables-to-be-same/
